# Assessment of technological options and economical feasibility for cyanophycin biopolymer and high-value amino acid production

**DOI:** 10.1007/s00253-007-1178-3

**Published:** 2007-09-18

**Authors:** Hans Mooibroek, Nico Oosterhuis, Marco Giuseppin, Marcel Toonen, Henk Franssen, Elinor Scott, Johan Sanders, Alexander Steinbüchel

**Affiliations:** 1 Wageningen University and Research Centre, Chair of Valorization of Plant Production Chains, P.O. Box 17, NL-6700 AA Wageningen, The Netherlands; 2 Department of Biobased Products, Agrotechnology and Food Sciences Group, Wageningen University and Research Centre, P.O. Box 17, NL-6700 AA Wageningen, The Netherlands; 3 Easthouse Business Solutions B.V., Landschrijverlaan 35, NL-9451 KT Rolde, The Netherlands; 4 AVEBE B.A., P.O. Box 15, NL-9640 AA Veendam, The Netherlands; 5 Wageningen University and Research Centre, Plant Breeding, P.O. Box 386, NL-6700 AJ Wageningen, The Netherlands; 6 Wageningen University and Research Centre, Molecular Biology, P.O. Box 8128, NL-6700 ET Wageningen, The Netherlands; 7 Institute for Molecular Microbiology and Biotechnology, Westfälische Wilhelms-Universität Münster, Corrensstraße 3, 48149 Münster, Germany

**Keywords:** Biorefinery, Plant waste, rest stream, Protamylasse, Cyanophycin, Non-ribosomal, N-functionality, Bulk chemicals

## Abstract

Major transitions can be expected within the next few decades aiming at the reduction of pollution and global warming and at energy saving measures. For these purposes, new sustainable biorefinery concepts will be needed that will replace the traditional mineral oil-based synthesis of specialty and bulk chemicals. An important group of these chemicals are those that comprise N-functionalities. Many plant components contained in biomass rest or waste stream fractions contain these N-functionalities in proteins and free amino acids that can be used as starting materials for the synthesis of biopolymers and chemicals. This paper describes the economic and technological feasibility for cyanophycin production by fermentation of the potato waste stream Protamylasse™ or directly in plants and its subsequent conversion to a number of N-containing bulk chemicals.

## Introduction

Plants have the ability to use the incident sunlight for the biosynthesis of a tremendous variety of compounds that may contain a number of functionalized atoms or groups. An important group of these functionalized compounds are proteins and especially the individual amino acids that contain one or more nitrogen atoms. When starting from crude oil or naphtha, the incorporation of functionalities (e.g., −NH_2_) into derived bulk chemicals (such as 1,2-ethanediamine and 1,4-butanediamine) requires considerable amounts of energy and catalysts. However, some amino acids appear to be very suitable starting materials for highly functionalized bulk chemicals (Scott et al. 2007).

The biorefinery concept is a rapidly emerging field of research and commercial activities aiming at the integral use of all components of agricultural crops. In addition to the main product such as starch or oil, also, other side stream fractions including protein, free amino acid, and fiber fractions have high potential for valorization. An example of such waste stream fraction, Protamylasse™ that remains after starch and protein extraction from potato and its possible application as a substrate for microbial fermentation and production process for cyanophycin (Elbahloul et al. 2005a,b, 2006), will be described in detail in the current paper.

Cyanophycin (multiarginyl-poly[l-aspartic acid]; CGP, cyanophycin granule peptide) is a non-ribosomal protein-like polymer which consists of equimolar amounts of aspartic acid and arginine arranged as a poly-aspartic acid backbone to which arginine residues are linked to the β-carboxyl group of each aspartate by its α-amino group. In nature, cyanophycin is produced by most, but not all, cyanobacteria as a temporary nitrogen reserve material during the transition of cells from the exponential phase to the stationary phase. The polymerization reaction is catalyzed by only one enzyme, which is referred to as cyanophycin synthetase (CphA). Because of the low polymer content and the slow growth of cyanobacteria resulting in only low cell densities, cyanobacteria are not suitable for large-scale production of cyanophycin. Therefore, the *cphA* genes from a number of cyanobacteria have been expressed in several bacteria, and, more recently, also in plants. Furthermore, the polymer isolated from recombinant strains contained lysine as an additional amino acid constituent.

Now that cyanophycin can be produced in sufficient amounts by pilot scale fermentations for studying its material properties, it appears of biotechnological interest because purified cyanophycin can be chemically converted into a polymer with a reduced arginine content, which might be used like poly-aspartic acid as a biodegradable substitute for synthetic polyacrylate in various technical processes. In addition, cyanophycin might also be of interest for other applications when the hitherto unknown physical and material properties of this polymer will be revealed. On the other hand, cyanophycin is a convenient source of the constituent amino acids that may be regarded as nitrogen-functionalized precursor chemicals.

In the current paper, conditions will be discussed for the technological and economic feasibility of cyanophycin production by microbial fermentation and by cyanophycin production directly in plants. The conditions for fermentative cyanophycin production will be based upon the use of cheap substrates derived from agricultural waste streams and the possible cyanophycin production simultaneously with other fermentation products like ethanol. This aspect is denoted process integration.

## Biorefinery and its place in the production of chemicals

The depletion in fossil feedstocks, increasing oil prices and the ecological problems associated with CO_2_ emissions, are forcing the development of alternative resources for energy, transport fuels, and chemicals: the replacement of fossil resources with CO_2_ neutral biomass.

Potentially, biomass may be used to replace fossil raw materials in several major applications: heat, electricity, transport fuels, chemicals, and other industrial use. Each of these groups represents about 20% of the total fossil consumption in the industrialized countries (Oil Market Report of the International Energy Agency 2004). Large variations in the cost of these products at the wholesale level, based on their energy content, are evident (Table 1). When one considers the contribution to costs by the raw materials (expressed per GJ end product), large differences are also seen. Heat can be produced from coal for around 3 €/GJ due to utilizing inexpensive feedstocks with high conversion efficiency (about 100%), while the raw material costs for electricity is double (6 €/GJ) due to a conversion yield of about 50%. Most notable is the high raw material costs for chemicals. Here, expensive raw materials (oil) are used with low(er) conversion yields (Sanders et al. 2005, 2007).

**Table 1:** Different applications and contributions of biomass

Contribution	Integral cost prices (€/GJ end product)	Raw material cost fossil (€/GJ)	Percentage of total energy in the Netherlands (3.000 PJ) consumed per application (%)
Heat	4	3 (Coal)	±20
Electricity	22	6 (Coal)	±20
Transport fuel	10	8 (Oil)	±20
Average bulk chemicals	75	30 (Oil)	±20
Rest of industry			±20

To obtain a good net income for biomass, an effective biorefinery system is required for the separation of the harvested crop into fractions for use in (several of) these applications. These may be used directly as the desired product or undergo conversion by chemical, enzymatic, and/or microbial means to obtain other products. Biorefinery systems are well established for a number of crops. For example, soybeans are the raw materials for large biorefineries to produce oil (for biofuels), proteins and valuable nutraceuticals.

Less well explored is the use of biomass to make industrial chemicals. Effort to produce chemicals with constant quality and performance (such as lactic acid) has been addressed, but has mainly focused on the use of carbohydrates as raw materials and use of biotechnology for conversion. However, for effective biorefinery approaches, other biomass fractions should also be considered for the production of chemicals. It is anticipated that the substitution of petrochemical transportation fuels with biofuels will rise significantly in the coming years. This means that a rise in the production of biodiesel will lead to large volumes of glycerol as a residual stream, and indeed, some companies are already investigating the use of glycerol to produce chemicals. While the awareness of large volumes of glycerol from biofuel production is apparent, one should not overlook other waste streams. Indeed, from biofuel production, an immense concomitant waste stream of protein will also be generated. Sources of proteins and amino acids are not limited to those generated from biofuel production, but also from other industries such as potato starch production. For example, during AVEBE’s processing of potatoes for starch extraction, the main waste stream is Protamylasse™, which mainly contains sugars, organic acids, proteins, and free amino acids and has currently no major market use. Some of the amino acids present in such sources could be very suitable raw materials for preparing (highly) functionalized chemicals traditionally prepared by the petrochemical industry (Sanders et al. 2007).

Generally, the conversion of crude oil products utilizes primary products (ethylene, etc.), and their conversion to materials or (functional) chemicals makes use of co-reagents such as ammonia and various process steps to introduce functionalities such as −NH_2_. Conversely, many products found in biomass, such as proteins and amino acids, often contain these functionalities. Therefore, it is attractive to exploit this to bypass the use, and preparation, of co-reagents as well as eliminate various process steps by utilizing suitable biomass-based precursors for the production of chemicals. Thus, the production of chemicals from biomass takes advantage of the biomass structure in a more efficient way than the production of fuels or electricity alone and can potentially save more fossil energy than producing energy alone (Scott et al. 2007). When used in combination with environmentally sound production and processing techniques across the whole biomass production chain, i.e., from cultivation and harvest, its (pre)treatment and conversion to products, the use of biomass is considered a sustainable alternative to conventional feedstocks, which is reflected by sound economic advantages in both raw material and investment costs.

## General introduction on NRPs and especially cyanophycin

**Fig. 1:**
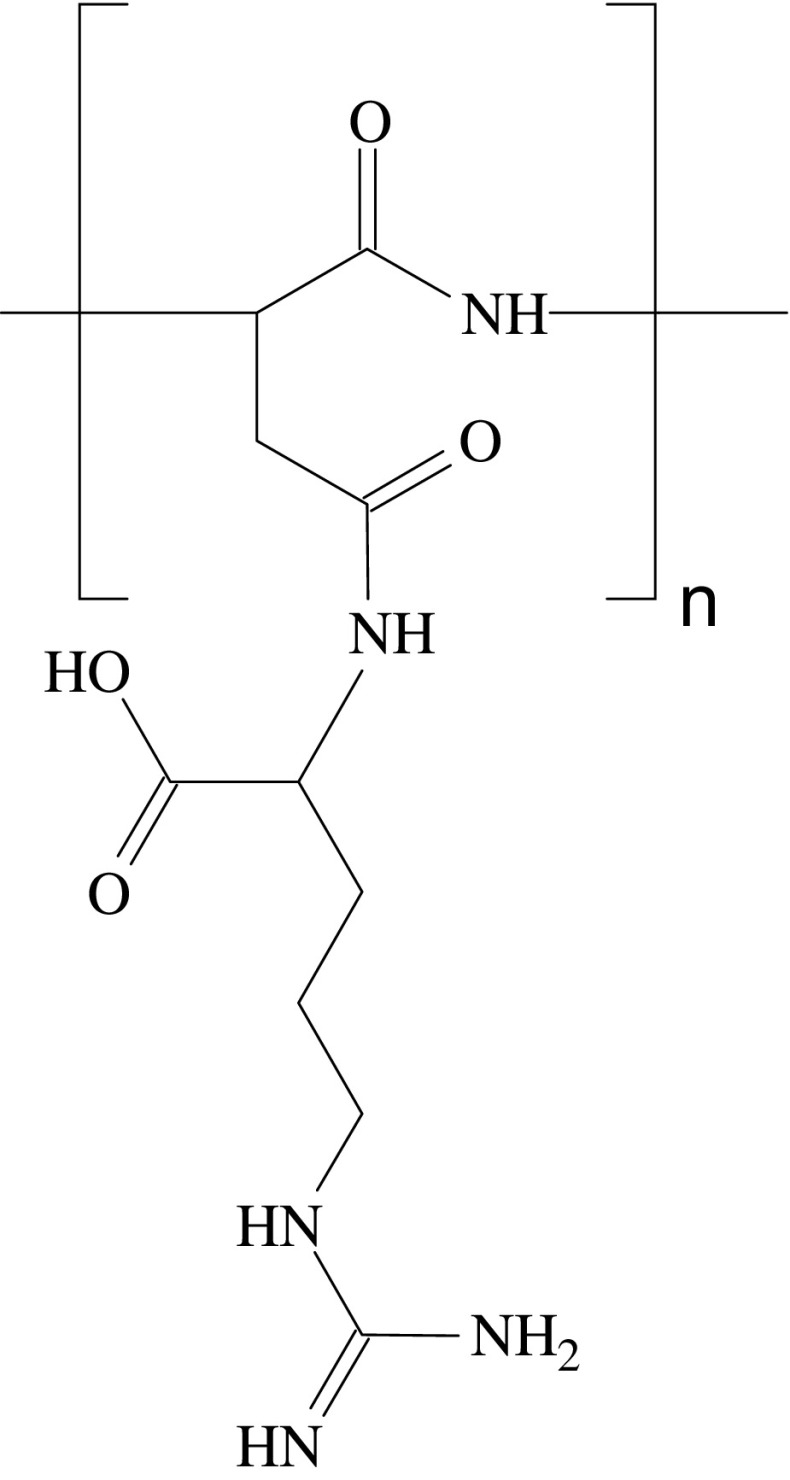
Chemical structure of the cyanophycin monomer

Cyanophycin (also referred to as CGP, cyanophycin granule polypeptide) that belongs to the family of bacterial poly-amino acids together with poly-γ-glutamic acid and poly-ɛ-lysine, was discovered in 1887 by Borzi (1887) during microscopic studies of cyanobacteria and was later found in all groups of cyanobacteria (Oppermann-Sanio and Steinbüchel 2002). The cyanophycin molecule structure is related to that of poly(aspartic acid)s, but, unlike synthetic poly-aspartic acid, it is a comb-like polymer with α-amino-α-carboxy-linked l-aspartic acid residues representing the poly(α-l-aspartic acid) backbone and l-arginine residues bound to the β-carboxylic groups of aspartic acids (Simon and Weathers 1976; for recent review, see Obst and Steinbüchel 2004; Fig. 1). Cyanophycin is synthesized by most, but not all, cyanobacteria as a temporary nitrogen reserve material during the transition of cells from the exponential phase to the stationary phase (Mackerras et al. 1990). At neutral pH and physiological ionic strength, cyanophycin is insoluble and deposited in the cytoplasm as membraneless granules (Lawry and Simon 1982).

Cyanophycin isolated from *Cyanobacteria* is highly polydisperse and shows a molecular weight range of 25–100 kDa as estimated by sodium dodecylsulphate polyacrylamide gel electrophoresis corresponding to a polymerization degree of 90–400 (Simon 1971; Simon and Weathers 1976). Cyanophycin is a transiently accumulated storage compound which is synthesized under conditions of low temperature or low light intensity. Its accumulation can be artificially enhanced by the addition of chloramphenicol as an inhibitor of ribosomal protein biosynthesis (Simon 1973). Cyanophycin plays an important role in the conservation of nitrogen, carbon, and energy and, as indicated by its biosynthesis in presence of chloramphenicol, is non-ribosomally synthesized by CphA. Cyanophycin is accumulated in the cytoplasm of cyanobacteria as membraneless granules (Allen and Weathers 1980) in the early stationary growth phase (Mackerras et al. 1990; Liotenberg et al. 1996). When growth is resumed, for example due to a change in cultivation conditions, cyanophycin is reutilized by the cells (Mackerras et al. 1990). Krehenbrink et al. (2002) and Ziegler et al. (2002) showed that cyanophycin occurs even in heterotrophic bacteria like *Acinetobacter* sp. and *Desulfitobacterium hafniense* and therefore confirmed the wide distribution of this biopolymer and its function in nature as a general storage compound.

Cyanophycin is of biotechnological interest because the purified polymer can be chemically converted into a polymer with reduced arginine content (Joentgen et al. 1998), which might be used like poly-aspartic acid as a biodegradable substitute for synthetic polyacrylate in various technical processes. In addition, cyanophycin might also be of interest for other applications if the unknown physical and material properties of this polymer are revealed. Because of the low polymer content and the slow growth of cyanobacteria resulting in only low cell densities, cyanobacteria are not suitable for large-scale production of cyanophycin (Schwamborn 1998), and sufficient amounts of cyanophycin were hitherto not available.

The polymerization reaction is catalyzed by only one enzyme, which is referred to as CphA (Ziegler et al. 1998). The *cphA* genes from *Anabaena variabilis* ATCC 29413, *Anabaena* sp. strain PCC7120, *Synechocystis* sp. strain PCC6803, *Synechocystis* sp. strain PCC6308, *Synechococcus elongatus*, *Synechococcus* sp. strain MA19 and others were cloned and expressed in *Escherichia coli* (Aboulmagd et al. 2000; Berg et al. 2000; Hai et al. 1999; Oppermann-Sanio et al. 1999; Ziegler et al. 1998). More recently, heterologous expression of *cphA* was also demonstrated at a small scale in recombinant strains of *Ralstonia eutropha*, *Corynebacterium glutamicum*, and *Pseudomonas putida* (Aboulmagd et al. 2001). Whereas in cyanobacteria the molecular mass of the polymer strands ranged from 25 to 100 kDa (Simon 1976), the polymer from recombinant strains harboring *cphA* as well as in vitro-synthesized polymer exhibited a much lower range (25 to 30 kDa) and polydispersity. Furthermore, it was found that the polymer isolated from recombinant strains contained lysine as an additional amino acid constituent (Aboulmagd et al. 2001; Ziegler et al. 1998). Recently, the results of a detailed *in silico* analysis of the occurrence of enzymes involved in cyanophycin metabolism was published (Füser and Steinbüchel 2007).

Recently, the earlier postulated instability of recombinant *E. coli* strains employed for cyanophycin production was also confirmed in both DH1 and DH5α. This instability may be caused by loss of the plasmid during fermentation. However, as cyanophycin production continues in cultures that rapidly appear to loose the ampicillin resistance employed for selection and plasmid maintenance, other explanations are also under consideration such as competition for Arg and Asp by both cyanophycin and the ampicillin resistance protein and the theoretical possibility that the ampicillin resistance protein could be trapped into the cyanophycin granule or at least be made inaccessible to the ampicillin.

Due to the wide knowledge of its metabolism and available genetic tools, *E. coli* is one of the most commonly used bacterial hosts for the production of recombinant proteins (Lee 1996). Several expression systems have been developed for technical-scale production of recombinant proteins in *E. coli* based on the regulated *trp*, *lac*, or lambda P_L_ promoter (Hannig and Makrides 1998). The cultivation of recombinant *E. coli* strains harboring *cphA* from *Synechocystis* sp. strain PCC6803 at the 500-l scale for the production of cyanophycin has been described (Frey et al. 2002). As the previously described method for the purification of cyanophycin (Simon and Weathers 1976) is not applicable to a large scale, a simplified method for isolation of the polymer at the technical scale was elaborated.

Biosynthesis of cyanophycin was extensively studied in the 1970s by Simon and coworkers (Simon 1971, 1976; Simon and Weathers 1976). Later, this led to the identification of cyanophycin synthetase enzymes and the encoding genes (*cphA*) in various organisms (Ziegler et al. 1998; Aboulmagd et al. 2000; Berg et al. 2000; Hai et al. 2002). Subsequently, the enzymes involved in the degradation of cyanophycin by intracellular cyanophcyinases of cyanobacteria (*cphB*) and extracellular depolymerases, like hydrolase (*cph*E) and cyanophycinase (*cph*I) genes, were identified (Obst et al. 2002, 2004; Obst and Steinbüchel 2004). Elbahloul et al. (2005a,b) have found that inactivation of the cyanophycinase gene in *Acinetobacter* resulted in significantly less cyanophycin accumulation than the wild type presumably due to a shortage of cyanophycin primer molecules. On the contrary, cyanophycin is highly resistant against hydrolytic cleavage by proteases such as trypsin, pronase, pepsin, carboxypeptidases B, carboxypeptidase C, and leucin-aminopeptidase, and cyanophycin is also resistant against arginases (Simon and Weathers 1976).

Because the cyanophycin synthetase genes (*cphA*) of many cyanobacteria, and recently, also of other microorganisms were identified, cloned, and heterologously expressed in other bacteria (Aboulmagd et al. 2001) conferring the ability to produce comparably large amounts of cyanophycin (up to 50% of CDW) in a much shorter period of time (1–2 days) as compared to cyanobacteria (about 4 weeks) and cyanophycin production was demonstrated at the 30- to 500-l scale (Aboulmagd et al. 2001; Frey et al. 2002; Voß and Steinbüchel 2006), the interest in cyanophycin as a potential raw material has constantly increased over the last few years. Improvement of fermentation conditions, feeding regimes, and the possibility to produce cyanophycin now by the employment of many genetically engineered bacteria with industrial relevance like *R. eutropha*, *C. glutamicum*, or *P. putida* (Aboulmagd et al. 2001) in complex but also in defined media make it appear likely that further improvement of cyanophycin production in bacteria will be achieved during future studies. These studies may include elementary mode analyses (Diniz et al. 2006) or more conventional approaches involving experimental design using fermenter arrays and principal component analyses.

In conclusion, large-scale fermentation processes for cyanophycin production and downstream processing are available for a number of different microorganisms able to grow in different substrates, including the potato waste stream Protamylasse™, and also, low cyanophycin yields were reported in plants.

## Production and economic aspects of fermentative cyanophycin production

An important contribution to sustainability can be made by the use of a considerable plant waste stream for the production of renewable, biodegradable, and biocompatible polymers and/or valuable chemicals that are now produced on large scale from petroleum. Some of the polymer classes to be developed may be expected to replace some existing mineral petroleum-based polymers as soon as competitive production prices can be obtained and/or supporting measures will be taken to promote the use of renewable resources. On the other hand, completely novel types of biopolymers may be developed for completely novel applications.

AVEBE, located in the northern part of The Netherlands, is the largest potato-starch-producing company in the world involved in extraction, processing, and sales of starch and starch-derived products. During processing of potatoes for starch extraction, the main waste stream is Protamylasse™. Annually, AVEBE produces about 120,000 m^3^ of Protamylasse™ containing about 70,000 tons of dry matter, mainly consisting of sugars (14,000 tons), organic acids (13,300 tons), proteins and free amino acids (18,000 tons). The amino acids arginine and aspartic acid amount to about 1,000 tons each and lysine to about 700 tons. Currently, there is no proper outlet for this Protamylasse™ other than low value epandage (e.g., by Bos Agra-Service, NL in which the salts present, such as potassium, are used as fertilizer), whereas rough calculation shows that the total intrinsic gross value of the valuable components in the Protamylasse™ is about 45 million euros. A number of research objectives is now in progress to add substantial value to the entire potato starch production chain (and thus its economical feasibility). Protamylasse™ may be considered a model for other agricultural waste streams, such as grass juice and beet residue. It is not certain whether concentrations of all medium components in the Protamylasse™ will be optimal to sustain microbial growth and cyanophycin production, and it is anticipated that additional medium components need to be identified and/or tailor-made production strains constructed. This will require a detailed analysis of Protamylasse™ components before and after the cyanophycin production phase.

To calculate the economic feasibility of cyanophycin production using Protamylasse™, several aspects need to be included as follows:

- Necessary steps from potato starch and protein extraction, yielding Protamylasse™, to the final purified product cyanophycin (which is considered an intermediate product for derived polymer types and N-containing bulk chemicals) include: shipment to a fermentation plant, dilution to 5–6% (*v*/*v*) using tap water, removal of potato particles by filtration, disposal (or alternative use) of particle fraction (60% DM), loading of step up fermenters [to allow an microbial inoculum concentration of 10% (*v*/*v*) per step; e.g., 10→100→1,000→10,000→100,000 l], sterilization, cooling, addition of ampicillin, inoculation, (batch) fermentation, addition of acid and/or base for pH control, cell harvesting and concentration, disposal or recycling of spent Protamylasse™, cell disruption (not for *E. coli*), cyanophycin extraction using pH 2, neutralization, cyanophycin crystallization, precipitation, purification, and storage.
- Yearly Protamylasse™ supply, 120,000 m^3^; 70,000 tons (60% DM)
- Protamylasse™ dry matter composition: amino acids (18,000 tons), sugars (14,000 tons), organic acids (13,300 tons), ash (22,200 tons). For further details on the composition, see Elbahloul et al. (2005a, b).
- Small-scale fermentation process data: Protamylasse™ concentration, 5–6% (*v*/*v*); fermenter volume, 25 l; strain *E. coli* DH1 (pMa/c5-914::*cphA*); temperature 37°C; maximal OD and cyanophycin yield reached after 15 h; optimal pH 7.5–8.0; biomass yield 5–10 g/l (CDW); cyanophycin content, 25% (*w*/*w*); cyanophycin composition, Asp, 50%; Arg, 45%; Lys, 5%; *E. coli* occasionally (5–10%) incorporates lysine in stead of arginine; poly(Asp–Arg) is non-soluble in water at neutral pH; poly (Asp–Arg–Lys) is soluble in water.

Assuming that most of the solid particles will be removed from the Protamylasse™ by filtration, a yearly amount of 48,000 m^3^ of Protamylasse™ liquid juice will become available, which is used at a 5% dilution during fermentation, thus, providing a yearly amount of 960,000 m^3^ of diluted fermentation broth. This volume would be enough to run 9,600 × 100 m^3^ fermenter volumes. Further assuming a 1 week’s run time (including cleaning, sterilization, fermentation, and harvest) a park of, e.g., 185 × 100 m^3^ or preferably 20 × 1,000 m^3^ fermenter units (step up units included) could be operated continuously. With the current *E. coli* biomass yield of 5 g/l (CDM) with a cyanophycin content of 25% (*w*/*w* DM), this would yield a yearly amount of 1,200 tons of purified cyanophycin. At an estimated market price of € 1,000 per ton cyanophycin, the Protamylasse™ juice fraction (40% *v*/*v*) would yield a yearly income of only 1.2 million euros. However, by only increasing the amount of microbial biomass from the actual 5 g/l (CDM, *E. coli*) to a realistic value of 100 g/l (CDM, *S. cerevisiae*) with the same cyanophycin content of 25% (*w*/*w* DM), this yearly income could be raised to 24 million euros. If within the same fermentation run via so-called process integration, in addition to cyanophycin, ethanol also [yielding from 960,000 m^3^ at 5% (*v*/*v*) about 48,000 m^3^ ethanol and an additional yearly income of 27.4 million euros at $2.8 per US gallon, € 0.57 per liter) could be produced using semi-aerobic fermentation with *S. cerevisiae*, this yearly income could be further raised to about 50 million euros. The Protamylasse™ particle fraction can, in principle, also be used for cyanophycin and ethanol production. Preliminary calculations using the model of Golden Grain Energy, LLC (http://sec.edgar-online.com/2004/06/14/0001104659-04-016859/Section7.asp) suggest that the break-even point could roughly be reached within about 3–5 years.

Further process improvements may be obtainable and are necessary to make this process economically feasible. These obtainable figures provide estimates as to the investments and costs that will be necessary to start a cyanophycin production fermentation facility. However, before fermentative cyanophycin production on industrial scale can be started, a number of bottlenecks should be overcome. Table 2 lists current bottlenecks and proposed measures for optimal and economically feasible cyanophycin production.

**Table 2:** Economical and technological bottlenecks and proposed measures

Bottleneck	Proposed measure(s)
Investments, including costs for fermentation and downstream processing equipment	The calculation provided here suggests that these may be acceptable
Costs for the production of cyanophycin, cyanophycin-derived products and for downstream processing of biomass	Construction of a sufficiently productive microbial strain to convert or simply utilize constituents of plant waste streams like Protamylasse™ and to incorporate these compounds, presumably amino acids, into the cyanophycin polymer chain during cyanophycin biosynthesis
Phenotypic instability of *E. coli* production strains used until now, DH1 and DH5α, containing plasmid pMa/c5-914::*cphA*_6803_	Construction of stable strains with integrated copies of the cyanophycinsynthesis genes
Low biomass yields of the *E. coli* strains used	Since not all components present in the current source of Protamylasse™ may have the proper concentration for current laboratory strain(s), an optimization may require the addition of substrates other than Protamylasse™, for example other plant waste streams. Sufficient provision of amino acids like arginine should be ensured during the production phase
Optimization of microbial biomass formation	By using yeasts as alternative production organisms biomass yields could be increased to 100 g/l CDM for *S. cerevisiae* [factor 20×] or 150 g/l CDM for *Pichia pastoris* [factor 30×] if in Protamylasse™ the same yields can be obtained as in dedicated growth media
Sub-optimal fermentation processes	Fermentation technology and feeding regimes have to be developed for optimum amino acid utilization or biosynthesis from Protamylasse™ or other plant waste streams
Generation of valuable side stream particle fraction of Protamylasse™	Alternative use of the side stream particle fraction of Protamylasse™, e.g. by using cyanophycin producing filamentous fungi
Co-production with, e.g., ethanol	When using *S. cerevisiae* as the production organisms and (semi-) anaerobic fermentation both cyanophycin and ethanol could possibly be produced during the same run
Costs for cyanophycin extraction	Development of alternative cheap cyanophycin extraction methods using, e.g., hydro-cyclone equipment for the non-soluble fraction
Cost-efficient production of cyanophycin in plants	The transfer of the bacterial cyanophycin synthetase gene (*cphA*) into eukaryotic hosts, mostly plants and its effective expression in suitable organs or cell compartments is a major step (see below)
Efficacy of downstream processing	Downstream processing has to be adapted and optimized for cyanophycin or cyanophycin derivatives containing biomass, which will be either bacterial cells or eukaryotic (mostly plant) cells or tissues
Lack of insight in possible modifications of cyanophycin, their impact on cyanophycin properties and market potential	The diverse possibilities to modify the cyanophycin molecule chemically or enzymatically has to be exhaustingly explored to identify all potential key applications for cyanophycin-derived products and to find the most suitable products with regard to market potential and the possibility of their commercialization
Lack of knowledge concerning properties of known cyanophycin synthetases and their genetic engineering	The possibility to modify the active sites of the cyanophycin synthetases in order to change its substrate specificity and to allow the production of cyanophycin derivatives has to be determined
Insufficient insight in all possible applications for cyanophycin as a polymer or as a starting material for chemical syntheses	The exploitation of cyanophycins and cyanophycin-derived molecules as substitutes for well established industrial products or as renewable raw materials has to be determined precisely

## Cyanophycin production in plants

Transgenic plants can be utilized to produce renewable resources for industrial purposes in a CO_2_-neutral, environmentally acceptable, and competitive way. Poly-3-hydroxybutyrate (PHB) was the first plastic-like compound produced in plants (Poirier et al. 1992), followed by, e.g., poly-3-hydroxyalkanoate (PHA; Poirier 2002) and medium chain-length PHA in potato (Romano et al. 2003, 2005) and showed the feasibility to produce biopolymers in plants. Recently, it has been shown that it is also possible to produce cyanophycin in plants (Neumann et al. 2005). For this, the *Thermosynechococcus elongatus* BP-1 cyanophycin synthetase gene was expressed constitutively under a 35S promoter in tobacco and potato plants. It was shown that approximately 1.14 and 0.24% dry weight could be accumulated in the cytosol of tobacco and potato leaves, respectively. The size (35 kDa), amino acid composition (Asp/Arg/Lys = 1:1.05:0.1), and structure of the plant-produced polymer was similar to that produced in transgenic *E. coli* expressing the same gene; however, the amount and molecular weight of the cyanophycin produced in plants was much lower than that observed in bacteria (up to 50% dry weight and 125 kDa in bacteria). The experiments have provided proof of concept for the potential of producing cyanophycin in plants.

Production of the cyanophycin biopolymer in potato is of high interest to the potato starch industry. Production in this plant does not require any additional infrastructure. After processing of the potatoes, cyanophycin can be isolated from the Protamylasse™. However, for commercial application, the efficiency of cyanophycin accumulation in potato has to be significantly improved.

Neumann et al. (2005) already indicated that directing the cyanophycin synthetase into several other compartments, such as the chloroplasts, could lead to increased accumulation of cyanophycin. However, chloroplasts in the cyanophycin-producing cells differ morphologically from wild-type chloroplasts. There are fewer and smaller grana stacks, and the growth rate is slower. One of the possible explanations for these properties is depletion of amino acid resources as a result of cyanophycin production (Neumann et al. 2005). The pioneering experiments by Neumann et al. (2005) have opened up a new field for the production of cyanopycin in agricultural crops and show that more research is needed before being introduced into agricultural production (Conrad 2005).

## Additional strategies

### Priming cyanophycin elongation

In *in vitro* studies, it has been shown that cyanophycin synthetase works more efficiently with a (β-Asp/Arg)_3_/Arg primer. *In planta* production of such a primer may enhance cyanophycin biosynthesis. Cyanophycinases encoded by the *chpB* and *chpE* genes can degrade cyanophycin into arginine–aspartic acid dipeptides (Asp–Arg) which cannot be used as primer for cyanophycin biosynthesis.

The *cphI* gene encodes a plant-type asparaginase able to hydrolyze β-Asp/Arg bonds and that, thus, may be responsible for the last step in cyanophycin degradation. Bacterial studies have indicated that *cphI* expression contributes to a higher cyanophycin level.

It might be possible to use a poly-Asp backbone as primer for cyanophycin biosynthesis. This peptide can be produced by ribosomal protein biosynthesis. The gene should be under the control of a low-level promoter to prevent the production of many peptides, and thus, the production of many low molecular weight polymers.

### Optimization of amino acid biosynthesis

It has been shown that cyanophycin synthetase uses arginine and aspartic acid as its major amino acid source but that it also can incorporate lysine in the cyanophycin polymer (Berg et al. 2000). It is unclear how this affects the properties of the polymer. Based on the chemical composition of Protamylasse™ and the affinity of the enzyme for arginine and lysine, it can be proposed that lysine accounts for 1.5% of the total cyanophycin. To reduce this amount, three strategies are possible, i.e., improve biosynthesis of arginine, reduce the level of lysine, and/or transform lysine into arginine.

It is possible that the availability of substrates (Asp and Arg) in plants is limiting or off-balance. Therefore, it is important to identify the organs that have the highest concentrations of available substrates and to investigate whether the substrate supply can be enhanced by introduction of genes involved in substrate production.

## Comparison of economics of cyanophycin production by fermentation or in plants

**Fig. 2:**
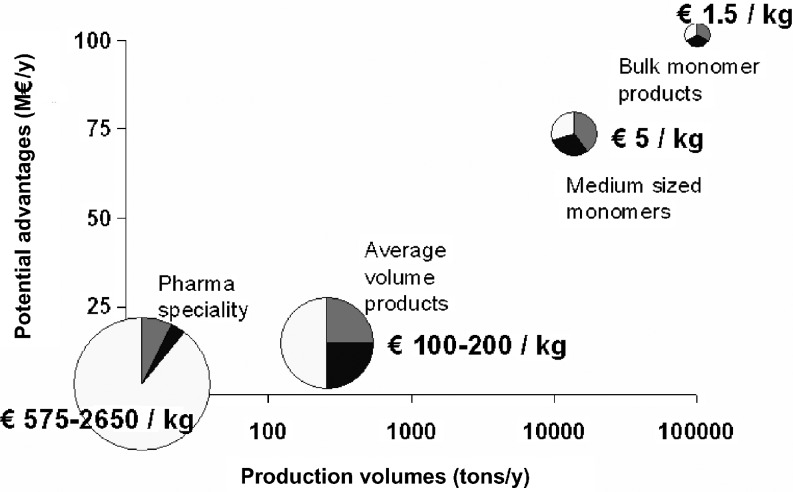
Cyanophycin production *in planta* or by fermentation. *Gray square*: raw material costs, *filled square*: fermentation costs, *open square*: recovery and purification costs

As for some other commodities, depending on production price, market volume and final product price (Fig. 2) for cyanophycin specialty product applications, fermentation is the preferred production method (roughly below 20.000 tons annually), whereas for bulk quantities of cyanophycin, the production directly in plants is preferred (above 20,000 tons). Therefore, for the production of cyanophycin-derived bulk quantities of nitrogen-containing chemicals, plants are considered the best production organisms, whereas for specialty polymers, fermentation may be the preferred production technology.

Assuming that a typical fermentative production of a bulk product, such as lysine, citric acid or glutamic acid costs about € 1,500 per ton and that these costs consist of: € 500 for the raw materials, € 500 for the fermentation process, and € 500 for recovery and purification. The advantage of producing in plants is that both the raw material costs and the fermentation costs can almost be neglected. On the other hand, recovery costs could be much higher. For the sake of the reasoning, it is assumed that recovery costs for cyanophycin production in plants will be the same as in the case of fermented production, i.e., € 500 per ton. In case of a fermentation process, a typical production volume for a company would be in the order of magnitude of 100,000 tons/year. The turnover would then be 150 million euros per year (i.e., € 1,500 per ton × 100,000 tons/year). In the case of production in plants, as raw material and fermentation costs are negligible, cost savings would be 100 million euros per year. This would be the maximum advantage, as this calculation does not include any costs for the production of the crop nor any additional costs for biorefining of the crop and treatment of side products. A similar reasoning would bring a maximum advantage for a product with a typical company production volume of 20,000 tons per year, such as the case with a medium-sized monomer. In this case, cost savings per ton would be higher, but as the volume is much smaller, the maximum advantage could be around 70 million euros. For specialty products with a volume of 300 tons per year (enzymes), a maximum advantage is estimated to be in the order of 5–10 million euros. For pharmaceutical production with a volume of 10 tons per year, the advantage would be around 0.5–1.5 million euros, as the volume would be small and production costs would mainly be ascribed to recovery.

## Other plant side streams

In addition to the possible exploitation of Protamylasse™, a large number of other plant side streams may be used for cyanophycin production, including grass juice, which remains after protein extraction or beet or cane molasses remaining after sugar extraction. It is foreseen that with the production of biodiesel and bioethanol, large volumes of side streams will become available, all containing major protein quantities. Such streams will include dried distillers grain and solubles (DDGS) from corn and wheat, press cake from palm oil and rape seed oil. These streams all have in common a very low cost price, which implies that when used as a microbial growth medium, a major contribution to fermentation process costs (about 30%) can be eliminated. However, it is important to note that none of any waste stream (to be) considered has exactly the same chemical or element composition as the production organism and its product(s), including, e.g., CO_2_. This implies that in each fermentation process to be developed using plant waste streams as substrates, limiting components should be supplemented. For the identification of such limiting components, fermentation test runs should be performed, preferably supported by statistic analyses such as elementary mode analysis (Diniz et al. 2006). Only after performing such exercises can reliable estimates of integral project costs be obtained.

## Cyanophycin-derived bulk chemicals

Cyanophycin can be hydrolyzed to its constituent amino acids, aspartic acid, and arginine. These amino acids may be utilized directly in food and pharmaceutical applications. However, based on the chemical structure of these amino acids and the presence of functionalized nitrogen-containing groups, it is possible to anticipate their conversion to a number of industrial chemicals, including:

- Arginine may be converted to 1,4-butanediamine. 1,4-diaminobutane, derived from petrochemistry, is currently used as a co-monomer in the production of nylon-4,6. The volume of production is not known, but is estimated to be in the range of 10,000s tons per year with a value of >€ 1,600 per ton.
- Conversion of aspartic acid to acrylonitrile is also envisaged. Using current petrochemical technology, the current worldwide market volume of acrylonitrile is 2.7–5 × 10^6^ tons per year and a price of about € 800–1,000 per ton.

Other chemicals which could be obtained from cyanophycin but are currently prepared from fossil resources include, e.g., 1,4-butanediol and urea.

The production of cyanophycin by plants will drastically reduce the cost price, potentially below € 1,000 per ton. This will enable the production of functionalized bulk chemicals such as 1,4-diaminobutane, and possibly, also acrylonitrile. The transition from mineral oil to plant-based precursor production has a considerable impact. The use of ammonia for the incorporation of nitrogen into chemicals is very important, but also very energy-intensive. Therefore, if the incorporation of nitrogen can be realized in systems based on plant (rest) streams in the form of protein or amino acid precursors, then this will yield considerable energy savings.

## Cyanophycin-based biopolymers

Poly-aspartic acid is derived from cyanophycin after the hydrolytic removal of arginine. This polymer has properties that are very similar to poly-acrylic acid. The cost price of this polymer can be set on 1,000€/ton in the cost calculations for arginine here above. As the volumes of these products will be similar, only 1,000 tons/year will be manufactured. Higher market prices might be obtained for special applications in food and/or pharmaceutical applications. This might change the cost structure of arginine in a way that market volumes might double or triple.

Cyanophycin as such might have applications as a polymer. Furthermore, derivatives obtained by enzymatic/genetic and/or chemical modifications might give valuable properties. Without thorough investigations, we cannot anticipate on the value of these polymers. Utilization in this area can be expected after 10 to 12 years after the beginning of the proposed research approaches for research and development.

## Possible cyanophycin modifications, applications for bulk chemicals and for polymers

By incorporating other amino acids, different types of polymers can be made. So far, several cyanophycins have been produced in recombinant strains of *E. coli* up to 50% dry weight. However, especially for health care medical and food packaging applications, *E. coli* may not be the best commercial production organism. Therefore, the development of alternative food-grade production organisms is also one of the objectives of the current activities. One suitable candidate may be the bakers yeast *Saccharomyces cerevisiae* (and others: see above). In addition, being stable at a pH between 3 and 9, cyanophycin can also be hydrolyzed in concentrated volumes into its pure components, arginine and aspartic acid. This would make the whole process a novel biological extraction procedure for the selected amino acids.

## Outlook

Given the anticipated cost development for fossil energy carriers and environmental regulations, the chemical industry is facing increasing financial pressure and is thus looking for possibilities to broach new resources as a basis for polymer production. Important considerations in this search are to lower energy costs and prices of raw material and to develop cheaper and more sustainable production processes.

Unlike poly-γ-glutamic acid and poly-ɛ-lysine, cyanophycin has not been commercialized yet. Cyanophycin can be broken down into the individual amino acids that can be used as building blocks in various industrial processes. Because of its homogeneous structure and composition, the cyanophycin polymer and its derivatives also appear to be good candidates as starting materials for the production of nitrogen-rich commodity products, which are based on nitrogen-rich chemicals, like, for example, nylons. For example, for poly(aspartic acid) which is the polymer backbone of cyanophycin, various applications have been developed ranging from water-softening or detergent applications to applications in the paper, building material, petroleum or leather industry, in cosmetics, as well as many dispersant applications.

Cyanophycin can be chemically converted into a polymer with a reduced arginine content, which might be used like poly-aspartic acid as a biodegradable substitute for synthetic polyacrylate in various technical processes (Schwamborn 1998). Thus, cyanophycin may find applications in cyanophycin-derived bulk chemicals and in cyanophycin-based biopolymers.

It can be expected that economical activities can be developed within the following areas, such as: fermentation industry, biopolymer production, processing, modification and product development (also for medical technology), packaging industry, food and feed supplementation industries and, last but not least, state-of-the-art technology (which, in turn, will attract additional financial sources and economical activities). It should be emphasized, however, that the mentioned applications are still uncertain and that these are so far only potential applications.

On the one hand, this development will lead to the substitution of chemicals that are now produced at the cost of fossil raw materials, such as oil. As oil may be depleted in about 50 years, and as there seems to be a correlation of the use of fossil raw materials with climate changes, it is essential to develop alternatives. The anticipated alternatives can be produced by fermentation and, in principle, by plant production systems, and so, giving a new economic and knowledge intensive value to the fermentation industry and/or to agriculture. On the other hand, novel types of polymers will be developed that do not simply replace existing applications but that will enter novel product markets.

Elements of the contents of this paper are included in a dedicated patent application (Elbahloul et al. 2006).
